# Computational Modeling of Parkinson’s Disease Across Scales: From Mechanisms to Biomarkers, Drug Discovery, and Personalized Therapies

**DOI:** 10.3390/brainsci16020175

**Published:** 2026-01-31

**Authors:** Sandeep Sathyanandan Nair, Aratrik Guha, Srinivasa Chakravarthy, Aasef G. Shaikh

**Affiliations:** 1 Department of Biomedical Engineering, Case Western Reserve University, Cleveland, OH 44106, USA; sandeep.nair@case.edu (S.S.N.); axg1334@case.edu (A.G.); 2 Cleveland FES Center, Louis Stokes Cleveland VA Medical Center, Cleveland, OH 44106, USA; 3 Daroff-Dell’Osso Ocular Motility Laboratory, Louis Stokes Cleveland VA Medical Center, Cleveland, OH 44106, USA; 4 Department of Biotechnology, Bhupat and Mehta Jyoti School of Biosciences, IIT Madras, Chennai 600036, India; schakra@ee.iitm.ac.in; 5 Department of Medical Science and Technology, IIT Madras, Chennai 600036, India; 6 Department of Neurology, University Hospitals, Cleveland, OH 44106, USA; 7 Department of Neurology, Case Western Reserve University, Cleveland, OH 44106, USA

**Keywords:** Parkinson’s disease, computational modeling, computational neuroscience, multiscale modeling, deep brain stimulation, L’DOPA, basal ganglia, therapeutics, eye movements, neurodegenerative disease

## Abstract

Parkinson’s disease (PD) is a multifactorial neurodegenerative disorder characterized by complex interactions across molecular, cellular, circuit, and behavioral scales. While experimental and clinical studies have provided critical insights into PD pathology, integrating these heterogeneous data into coherent mechanistic frameworks and translational strategies remains a major challenge. Computational modeling offers a powerful approach to bridge these scales, enabling the systematic investigation of disease mechanisms, candidate biomarkers, and therapeutic strategies. In this review, we survey state-of-the-art computational approaches applied to PD, spanning molecular dynamics and biophysical models, cellular- and circuit-level network models, systems and abstract-level simulations of basal ganglia function, and whole-brain and data-driven models linked to clinical phenotypes. We highlight how multiscale and hybrid modeling strategies connect α-synuclein pathology, mitochondrial dysfunction, oxidative stress, and dopaminergic degeneration to alterations in neural dynamics and motor and non-motor symptoms. We further discuss the role of computational models in biomarker discovery, including imaging, electrophysiological, and digital biomarkers. In particular, eye-movement-based measures are highlighted as quantitative, reproducible behavioral signals that provide principled constraints for individualized computational modeling. We also review the emerging impact of computational approaches on drug discovery, target prioritization, and in silico clinical trials. Finally, we examine future directions toward personalized and precision medicine in PD, emphasizing digital twin frameworks, longitudinal validation, and the integration of patient-specific data with mechanistic and data-driven models. Together, these advances underscore the growing role of computational modeling as an integrative and hypothesis-generating framework, with the long-term goal of supporting data-constrained predictive approaches for biomarker development and translational applications.

## 1. Introduction

Neurodegenerative diseases such as PD represent a major scientific and clinical challenge, affecting millions of individuals worldwide and imposing an increasing socioeconomic burden as populations age. PD is characterized by progressive neuronal dysfunction and loss, leading to irreversible cognitive and motor impairments [1]. Despite decades of intensive experimental and clinical research, effective disease-modifying therapies remain elusive, and diagnosis often occurs only after substantial neurodegeneration has taken place. A central obstacle to progress lies in the inherently multifactorial and multiscale nature of these diseases, which span molecular pathology, cellular vulnerability, circuit dysfunction, and behavioral impairment, challenging conventional reductionist approaches and prompting increased interest in integrative systems and computational frameworks.

At the molecular and cellular levels, PD involves protein misfolding and aggregation, mitochondrial dysfunction, oxidative stress, and impaired protein balance, ultimately leading to the selective degeneration of dopaminergic neurons in the substantia nigra pars compacta (SNc) and depletion of striatal dopamine [2]. At the circuit level, dopamine loss disrupts the dynamics of BG–thalamocortical loops, giving rise to pathological rhythms and impaired action selection that underlie hallmark motor symptoms and a range of non-motor manifestations [3]. At the behavioral level, beyond limb motor behavior, eye movement control provides a uniquely tractable domain for testing computational models of basal ganglia (BG) dysfunction, owing to its well-defined circuitry, rapid dynamics, and sensitivity to dopaminergic modulation [4,5,6]. Understanding how these processes interact across scales from molecular networks to whole-brain circuitry and behavior remains a foundational challenge for neuroscience.

Mathematical and computational models provide a powerful framework for integrating heterogeneous data and mechanistic hypotheses across levels of organization. Such models formalize biological processes in quantitative terms, enabling the systematic exploration of causal relationships, hypothesis testing, and prediction in ways that complement experimental and clinical studies. Modeling approaches have been applied across a broad spectrum that includes mechanistic models of pathogenic processes, multiscale network models of interacting molecular pathways, and dynamical models of BG circuitry and deep brain stimulation [3].

Beyond explanatory insight, these models are increasingly positioned as translational tools that can aid biomarker discovery, optimize therapeutic interventions such as deep brain stimulation, and explore patient-specific variability [3]. Recent developments in data acquisition, computational power, and integrative modeling methodologies are further accelerating the shift toward predictive and personalized neuroscience, suggesting new pathways toward digital twins and individualized therapeutic strategies.

In this review, we highlight how mathematical modeling serves as a conceptual and practical bridge across scales in neurodegenerative diseases, with PD as a primary focus. Throughout this review, we emphasize eye movement control as a principled behavioral assay for linking multiscale computational models to quantifiable clinical outcomes in Parkinson’s disease. We review recent modeling advances, discuss their contributions to understanding disease mechanisms and treatment strategies, and outline open challenges and future directions for predictive computational frameworks in neurodegeneration. This review discusses how such models now occupy a central position in neurodegenerative disease research and how they may shape the future of diagnosis and treatment.

## 2. Perspectives from Disease-Specific Modeling

PD is defined by the progressive degeneration of dopaminergic neurons in the SNc and the consequent disruption of BG–thalamocortical circuits, ultimately giving rise to abnormal action selection and motor behavior.

To provide a holistic perspective across scales, behavior is first considered at the level of actions within the framework of optimal control theory, in which behavior emerges from the selection of control policies that minimize expected costs while achieving task goals. Within this normative framework, reinforcement learning (RL) offers a computationally principled description of how value-based learning and action selection can be guided by reward prediction errors (RPEs). Dopaminergic signaling is commonly interpreted as encoding such errors, and loss of dopaminergic input from the SNc compromises learning, policy evaluation, and action selection under uncertainty.

At the circuit level, these behavioral impairments are accompanied by pathological network dynamics, most prominently exaggerated beta-band oscillations in the subthalamic nucleus (STN), globus pallidus externa (GPe), and related BG structures. Such dynamics are well captured by mechanistic spiking neuron models of BG circuits, which link dopamine depletion to abnormal synchrony and impaired action gating. At a deeper level, dopamine loss itself arises from cellular and molecular processes, including oxidative stress, mitochondrial dysfunction, protein aggregation, and α-synuclein pathology, which are addressed by biophysical cellular models of dopaminergic neuron vulnerability.

Together, these modeling approaches provide a multiscale framework that links abnormal actions in PD to circuit-level dynamics and, ultimately, to their underlying cellular and molecular mechanisms.

Computational models of PD operate at distinct levels of abstraction, ranging from normative or algorithmic frameworks that describe optimal decision-making principles to mechanistic circuit-level models that explicitly represent BG–thalamocortical connectivity, and further to biophysical cellular models that capture ionic, metabolic, and molecular processes. In this review, RL and optimal control models are treated as normative descriptions of behavior, whereas BG network and cellular degeneration models are considered mechanistic or implementation-level frameworks. Accordingly, we organize the subsequent sections into three broad categories—behavioral-level, circuit-level, and cellular-level models of PD. Mapping between these levels is conceptual rather than one-to-one, and care is taken throughout to distinguish explanatory abstractions from their biological realizations.

### 2.1. Behavioral-Level Models of PD

PD is characterized by a constellation of behavioral impairments, among which bradykinesia—the slowness in initiating and executing voluntary movements—stands as a defining clinical hallmark [7]. From a computational perspective, these impairments are increasingly understood not as failures of motor execution alone, but as disruptions in decision-making processes governing action selection, vigor, and effort allocation. This reframing has been driven by behavioral-level computational models that link dopaminergic signaling to normative principles of learning and control. Importantly, such models emphasize that BG dysfunction in PD affects not only motor behavior but also cognition, working memory, impulse control, and oculomotor function. This shift in perspective has motivated the use of RL frameworks, which offer a principled computational account of how dopaminergic signals shape learning and action selection.

#### 2.1.1. Dopamine, Prediction Errors, and RL

In classical RL formulations, agents learn action policies by iteratively updating value estimates based on RPEs, which are the differences between expected and actual outcomes [8]. Seminal computational and electrophysiological work established that phasic activity of midbrain dopamine neurons closely resembles such RPE signals [9,10]. This insight provided a principled computational interpretation of dopamine’s role in learning, positioning dopaminergic neurons as teaching signals that guide adaptive behavior. Within the BG, RL mechanisms are thought to be instantiated through cortico–striatal plasticity, with dopamine modulating synaptic updates in a manner consistent with temporal-difference learning [11]. Under healthy conditions, these mechanisms support efficient learning, rapid action initiation, and flexible adaptation to changing reward contingencies.

#### 2.1.2. Dopamine Loss and Learning Asymmetries in PD

If dopaminergic signaling plays a central role in RL under healthy conditions, its degeneration in PD is expected to systematically bias learning processes. In PD, the degeneration of dopaminergic neurons in the SNc leads to a substantial reduction in dopamine availability within the striatum. Computational models reproduce the experimentally observed phenomenon that this loss disproportionately impairs learning from positive outcomes while sparing or even enhancing learning from negative feedback. This pattern is consistent with empirical behavioral studies showing that unmedicated PD patients exhibit selective deficits in reward-based learning [12].

Neurocomputational models of the BG formalize this effect by distinguishing between D1 receptor-expressing “Go” pathways that facilitate action selection and D2 receptor-expressing “NoGo” pathways that suppress competing actions [13,14]. Dopamine depletion shifts the balance of this system toward excessive inhibition, resulting in conservative action policies, reduced exploration, and delayed movement initiation—computational signatures that map naturally onto bradykinesia and akinesia. While these learning asymmetries explain impaired action selection, they do not fully account for the pervasive slowing of movement observed in Parkinson’s disease.

#### 2.1.3. Action Selection, Vigor, and Opportunity Costs

Beyond learning, dopamine has been implicated in regulating the vigor with which actions are executed. Normative models propose that movement vigor reflects a trade-off between reward rate and energetic or temporal costs [15]. In this framework, tonic dopamine encodes the opportunity cost of time, biasing organisms toward faster or slower actions depending on the expected reward landscape.

Empirical and computational work has shown that PD patients behave as though the opportunity cost of time is underestimated, leading to slower movements even when faster actions would yield greater reward [7]. Related cost-based models of motor control further formalize bradykinesia as an increase in perceived effort or control costs, rather than a deficit in motor capability [16]. However, movement vigor is not expressed through discrete choices alone but through the continuous control of kinematic variables such as speed, force, and trajectory.

#### 2.1.4. From Discrete RL to Continuous Motor Control

While early RL models relied on discrete state and action spaces, biological motor control is inherently continuous, involving the graded control of force, velocity, and trajectory. To address this mismatch, computational frameworks have integrated RL with optimal control theory. Optimal feedback control models describe motor behavior as the minimization of task-relevant costs under uncertainty, producing flexible and efficient movement strategies [17].

A unifying computational framework was proposed in which the BG implements RL, the cerebellum supports supervised learning of motor dynamics, and the cortex performs state representation and planning [18,19]. Within this architecture, dopamine-modulated RL shapes policy selection in continuous action spaces, enabling smooth and adaptive motor behavior. Dopamine loss in PD disrupts this learning process, leading to slower convergence toward optimal control policies and increased movement variability, consistent with experimental observations in reaching and adaptation tasks [20].

Although these frameworks provide a normative account of motor behavior, linking them to PD requires grounding these computations in biologically realistic BG circuitry.

#### 2.1.5. Biologically Grounded BG Models

More recent models have incorporated detailed anatomical and physiological constraints of BG circuitry. Actor–critic architectures map naturally onto BG–thalamocortical loops, with the actor associated with striatal action selection and the critic linked to dopaminergic value prediction [11,21]. Extensions of these models include explicit exploration mechanisms and biologically plausible learning rules, such as the Go–Explore–NoGo (GEN) framework [22]. Traditionally, the BG was looked viewed as a Go–NoGo circuitry; however, the GEN framework, with STN–GPe acting as the exploratory engine [23], added a new dimension to PD modeling research.

A central strength of computational approaches lies in their ability to bridge levels of explanation. By mapping computational variables such as states, actions, policies, and prediction errors onto BG–thalamocortical loops [22,24], RL-based models provide a mechanistic account of how molecular dopamine loss gives rise to circuit dysfunction and, ultimately, clinical symptoms. However, this mapping is often described abstractly, and a clear conceptual correspondence between RL components and their biological substrates is not always made explicit.

**Figure 1:**
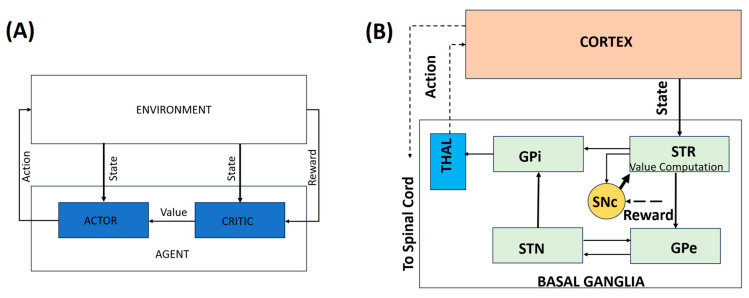
A representation of the behavioral model. (**A**) A simple RL-based model. (**B**) An illustrative mapping of the behavioral model to the BG–thalamocortical circuitry. Solid lines indicate local, explicitly modeled basal ganglia circuitry, whereas dotted lines represent indirect, polysynaptic, or abstracted pathways included for functional completeness.

Computational approaches to PD operate across multiple interacting scales, ranging from cellular pathology and dopamine signaling to BG dynamics and behavior. Rather than treating these levels in isolation, multiscale models explicitly link intracellular pathology, dopamine signaling, BG dynamics, and motor behavior, providing a unified framework for understanding disease progression and therapeutic intervention. Figure 1 addresses this conceptual gap by illustrating the correspondence between behavioral RL constructs and BG–thalamocortical circuitry. Panel A presents a generic RL architecture in which an agent interacts with its environment, while Panel B depicts its biological instantiation within the BG–thalamocortical loop. The mapping between the neural substrates and the corresponding RL components is summarized in Table 1. This is an illustrative mapping rather than a one-to-one neural implementation.

Overall, behavioral-level RL models have played a central role in advancing computational theories of PD. While they do not capture all aspects of disease progression, they provide a powerful explanatory framework for understanding how dopaminergic degeneration translates into impaired action selection, reduced motivation, and bradykinesia. When integrated with circuit-level and cellular models, RL-based approaches contribute critically to multiscale accounts of PD pathophysiology and support more integrative, mechanism-informed modeling across behavioral and neural scales.

Importantly, these models converge on the view that PD-related motor deficits reflect altered decision-making dynamics rather than pure motor impairments. Bradykinesia emerges as a rational consequence of abnormal cost–benefit computations, distorted learning signals, and overly inhibitory action selection policies. This computational reframing provides an explanatory framework that integrates motor and motivational symptoms of PD and helps account for the established effects of dopaminergic medication and deep brain stimulation (DBS).

Crucially, these computational principles are not restricted to motor behavior but generalize to cognitive domains that also rely on basal ganglia-mediated decision processes and other non-motor and autonomic domains.

#### 2.1.6. Extending BG Models Beyond Motor Control

While PD is classically defined by motor symptoms, computational models increasingly emphasize that BG dysfunction also affects cognition, working memory, and impulse control [25,26]. RL-based frameworks and generalized deep neural network agents have been proposed to capture a broad range of cognitive constructs, demonstrating that dopaminergic learning principles extend well beyond motor domains [27].

BG models of working memory show how dopamine-dependent gating mechanisms regulate information maintenance and updating, providing insight into cognitive deficits observed in PD [25,27,28]. Similarly, network models have identified circuit-level markers associated with medication-induced impulsivity, linking dopaminergic therapy to altered decision thresholds and risk-sensitive behavior. These studies reinforce the view that PD reflects a disorder of distributed decision-making systems rather than isolated motor circuits. Among the various behavioral domains affected by BG dysfunction, eye movement control offers a uniquely tractable and well-characterized testbed for linking computational models to measurable behavior.

#### 2.1.7. Eye Movement Control as a Computational Window into PD

Eye movement behaviors, including saccades, smooth pursuit, and fixation control, provide a powerful and well-characterized window into the neural mechanisms disrupted in PD. Unlike complex limb movements, eye movements are rapid, highly stereotyped, and supported by well-defined cortico–BG–brainstem circuits, making them particularly amenable to computational modeling. Patients with PD exhibit characteristic oculomotor abnormalities, including increased saccade latency, hypometric saccades, impaired smooth pursuit, and deficits in antisaccade tasks, reflecting disruptions in action initiation, inhibition, and reward-dependent learning [29,30]. These behavioral signatures closely parallel deficits accounted for by dopamine-modulated RL models, positioning eye movements as an ideal behavioral assay for linking computational mechanisms to BG dysfunction.

From a modeling perspective, eye movement control has played a foundational role in the development of internal model theories of motor learning and prediction. Computational frameworks proposed by Wolpert and colleagues emphasize the role of forward and inverse models in predicting sensory consequences of actions, while work by Shadmehr and collaborators highlights how motor learning emerges from error-based updating of these internal representations [31,32]. In PD, dopaminergic dysfunction and altered BG output are thought to impair the updating and selection of these internal models, leading to slowed initiation and reduced flexibility in oculomotor behavior. Integrating eye movement paradigms into RL and circuit-level models therefore offers a tractable route to link abstract learning variables, such as RPE, to measurable behavioral deficits and underlying neural dynamics.

### 2.2. Circuit-Level Models of PD

Building on behavioral-level RL frameworks that describe dopaminergic modulation of motor decision-making, circuit-level models provide mechanistic insight into the neural interactions within the BG that underlie pathological activity patterns in PD. These models move beyond abstract computations to capture how structural and synaptic connectivity, intrinsic neuronal properties, and dopamine depletion give rise to hallmark neurophysiological abnormalities, including excessive beta-band oscillations and impaired action selection.

Spiking network models further enhance biological realism by capturing the temporal dynamics of dopamine modulation and cortico-striatal plasticity [33,34]. These models reproduce key PD phenomena, including beta-band oscillations, reduced movement initiation, and impaired exploration, linking cellular-level pathology to system-level behavioral deficits.

#### 2.2.1. STN–GPe Dynamics and Beta Oscillations

A prominent circuit-level hallmark of PD is the emergence of exaggerated beta-band (13–30 Hz) oscillations within the basal ganglia, particularly in the STN and GPe [35]. These oscillations correlate with motor impairments such as bradykinesia and rigidity and are alleviated by dopaminergic medication and DBS [36]. Computational modeling studies have identified the reciprocal excitatory–inhibitory connectivity between the STN and GPe as a key and well-supported mechanism contributing to the generation and amplification of beta oscillations. Dopamine depletion alters the balance of excitation and inhibition within this loop, promoting synchronized firing and reducing the system’s capacity for flexible action selection [33,34].

At the same time, beta oscillations are increasingly viewed as arising from distributed BG–thalamocortical interactions, with contributions from cortical drive, thalamic dynamics, and potentially distinct beta sub-bands serving different functional roles [37,38,39]. The STN–GPe loop is therefore best understood as a central component within a broader network mechanism, rather than as the sole source of pathological beta activity [40,41].

#### 2.2.2. Action Selection and Behavioral Flexibility

Circuit-level models provide a mechanistic account of how dopamine-dependent deficits in RL translate into impaired motor exploration and action switching. In healthy states, the STN–GPe network facilitates dynamic suppression of competing motor programs and rapid switching between alternative actions, supporting flexible, goal-directed behavior [33,42]. In PD, dopamine depletion disrupts this regulatory function, leading to excessive synchrony, over inhibition, and reduced exploratory behavior—providing a computational account aligned with bradykinesia and reduced movement vigor characterized in RL-based behavioral models [7,15].

#### 2.2.3. Biophysically Realistic Spiking Models

Biophysically grounded models employ spiking neurons to capture tonic and burst firing patterns observed experimentally in STN and GPe neurons. The Izhikevich neuron model, in particular, offers a computationally efficient framework to reproduce the full spectrum of firing behaviors, from irregular tonic spiking to synchronized bursts, by adjusting a small set of parameters [43]. These models integrate excitatory glutamatergic inputs (NMDA/AMPA-mediated) and inhibitory GABAergic projections from neighboring neurons and the striatum, allowing the simulation of how dopamine depletion shifts excitation–inhibition balance, promotes pathological feedback loops, and stabilizes beta oscillations [33,44].

#### 2.2.4. DBS and Network Modulation

Circuit-level modeling has been instrumental in elucidating the mechanisms of therapeutic interventions, particularly DBS. High-frequency stimulation of the STN is known to suppress pathological beta oscillations and improve motor symptoms [45,46]. Computational models simulate DBS as an external modulatory input that perturbs STN–GPe network activity, demonstrating that therapeutic effects emerge not from simple inhibition but from the disruption of abnormal synchrony and the restoration of desynchronized, information-rich firing patterns [26,47,48]. These simulations provide mechanistic predictions for optimal stimulation parameters, electrode placement, and network-level effects of neuromodulation.

#### 2.2.5. Linking Circuit Dynamics to Behavioral Computations

Circuit-level models bridge the gap between behavioral RL deficits and neural physiology. By embedding STN–GPe interactions within the broader BG–thalamocortical loop, these models explicitly connect dopamine-dependent disruptions in action selection and learning to the emergence of pathological oscillations and excessive synchrony [33,35,42]. Importantly, they demonstrate how multiscale dynamics—from synaptic interactions to network oscillations—translate into observable motor impairments, providing a mechanistic substrate for behavioral phenomena such as bradykinesia, reduced movement vigor, and impaired exploratory behavior [7,15].

As discussed in the previous section, behavioral models capture deficits in action selection and learning but do not, by themselves, explain how these impairments emerge from specific circuit-level interactions within the basal ganglia.

**Figure 2:**
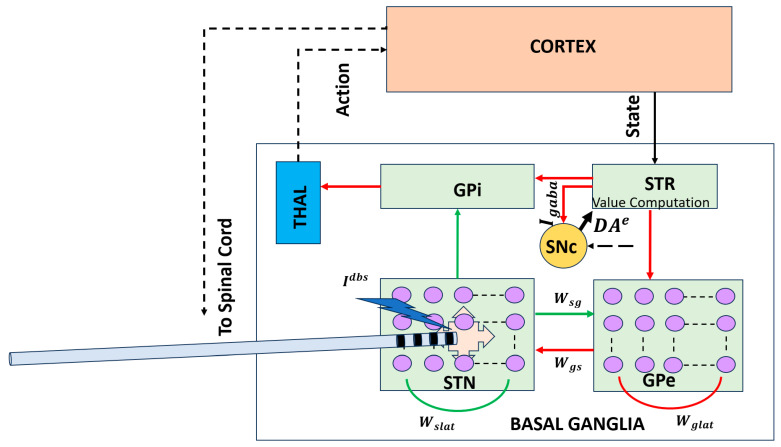
Circuit-level BG–thalamocortical model highlighting the STN–GPe loop and DBS. Pathological synchrony and beta oscillations emerge under dopamine-depleted conditions and are modulated by high-frequency stimulation [43]. Solid lines indicate local, explicitly modeled basal ganglia circuitry, whereas dotted lines represent indirect, polysynaptic, or abstracted pathways included for functional completeness. Red arrows denote inhibitory (GABAergic) connections, and green arrows denote excitatory (glutamatergic) connections. STR: striatum; THAL: thalamus; $W_{gs}$: synaptic weight from GPe to STN; $W_{sg}$: synaptic weight from STN to GPe.

Figure 2 addresses this conceptual gap by illustrating a circuit-level BG–thalamocortical model that explicitly represents interactions between the STN and GPe, a core BG subcircuit implicated in Parkinson’s disease. Dopamine depletion alters the excitation–inhibition balance within this loop, giving rise to excessive synchrony and pathological beta-band oscillations that impair flexible action selection. Incorporating DBS into this framework enables investigation of how high-frequency stimulation disrupts abnormal network dynamics and restores the desynchronized activity associated with improved motor function.

### 2.3. Cellular Models of Dopaminergic Degeneration

While behavioral and circuit-level models often represent dopamine as a modifiable signal in a neural network, cellular models focus on the intrinsic cellular processes that underlie dopaminergic degeneration in PD. These models are critical for understanding how dopamine-producing neurons in the SNc deteriorate over time due to cellular stress and the molecular mechanisms associated with PD pathology. Key mechanisms implicated in neurodegeneration include oxidative stress, mitochondrial dysfunction, calcium overload, and excitotoxicity [49,50,51].

It is important to emphasize that cellular models of dopaminergic degeneration do not attempt to replicate the absolute temporal scale of PD progression, which unfolds over years or decades in humans. Instead, they capture mechanistic trends and relative vulnerability patterns, enabling the investigation of how intracellular stressors and feedback interactions qualitatively drive progressive dopamine loss.

By embedding cellular models of SNc neurons within larger BG–thalamocortical networks, these models offer a dynamic framework for studying progressive dopamine loss as an emergent process rather than a static parameter. As dopamine depletion progresses over time, these models allow for simulations that reflect the heterogeneous and nonlinear nature of neurodegeneration, which is a hallmark of PD observed in both patients and experimental models.

#### 2.3.1. Mechanisms of Dopaminergic Cell Loss: Oxidative Stress and Mitochondrial Dysfunction

One of the key cellular mechanisms driving dopaminergic degeneration in PD is oxidative stress, which results from an imbalance between reactive oxygen species (ROS) and the cell’s ability to neutralize them with antioxidants [52]. Dopaminergic neurons are particularly vulnerable to oxidative damage because dopamine itself can undergo auto-oxidation, producing ROS that damage cellular components like proteins, lipids, and DNA. Computational models incorporating oxidative stress demonstrate how this damage triggers a cascade of intracellular events, including mitochondrial dysfunction, calcium overload, and ultimately cell death [51,53,54,55].

For example, computational simulations of SNc neurons highlight how sustained calcium influx through voltage-gated calcium channels leads to mitochondrial dysfunction by disrupting calcium buffering mechanisms, increasing energetic demand, and impairing ATP production [51,56]. This cascade results in the reduced synthesis of dopamine, which further exacerbates the deficit in dopaminergic signaling and accelerates the progression of PD.

#### 2.3.2. Calcium Dynamics and Cellular Vulnerability

At the cellular level, SNc neurons exhibit autonomous pacemaking activity that is heavily reliant on calcium dynamics. The pacemaker activity in these neurons is modulated by calcium channels, which are integral to their regular firing patterns [34]. Computational models that incorporate calcium-dependent processes can explain how sustained calcium influx under pathological conditions increases neuronal vulnerability. This leads to oxidative damage, impaired dopamine synthesis, and ultimately dopaminergic degeneration. These mechanisms, when modeled computationally, also illustrate how disruptions in normal calcium homeostasis underlie key motor impairments seen in PD, including bradykinesia and rigidity.

#### 2.3.3. Progressive Dopamine Loss and Feedback Dynamics

An important feature of cellular models is their ability to simulate progressive dopamine loss over time. By incorporating synaptic plasticity and intracellular stress responses into a BG network, these models can investigate how dopamine depletion reshapes network-level dynamics and motor behavior [48,55,57]. For example, cellular models of the SNc reveal how dysregulated signaling from the striatum can give feedback into the SNc, exacerbating cellular stress, and accelerating neurodegeneration. Striatal projections that influence SNc firing patterns form a closed-loop system, with altered motor and cognitive demands accelerating the degenerative process. This feedback mechanism is essential for understanding the heterogeneity of PD symptoms, as different brain regions and circuits are affected at varying rates [58,59,60].

**Figure 3:**
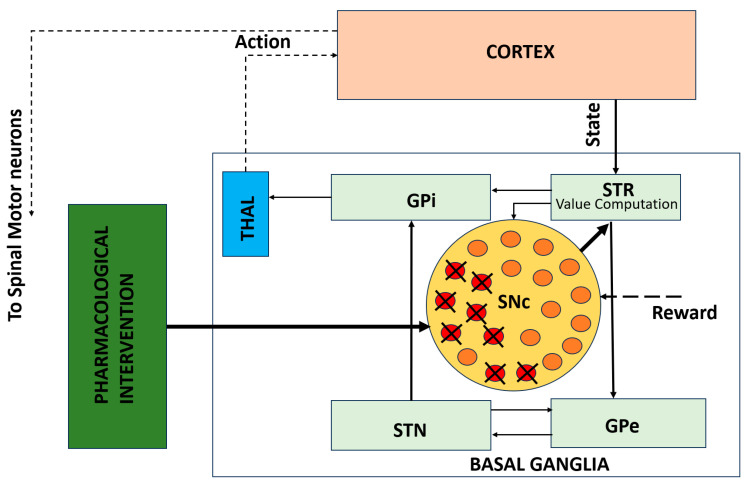
Cellular-level model of SNc degeneration embedded within the BG–thalamocortical network. The framework captures progressive dopamine loss driven by cellular stress mechanisms and allows simulation of pharmacological interventions [50]. Solid lines indicate local, explicitly modeled basal ganglia circuitry, whereas dotted lines represent indirect, polysynaptic, or abstracted pathways included for functional completeness.

While circuit-level models explain how network dynamics produce motor deficits, they do not capture the cellular mechanisms underlying progressive dopaminergic degeneration. Figure 3 illustrates this gap by depicting a cellular-level model of SNc neurons embedded within the BG–thalamocortical network. This framework incorporates progressive dopamine depletion, leading to altered dopamine signaling over time. Embedding these cellular processes within network-level models enables the investigation of how neuronal degeneration reshapes circuit dynamics and behavioral outcomes and how pharmacological interventions may modulate these effects.

**Figure 4:**
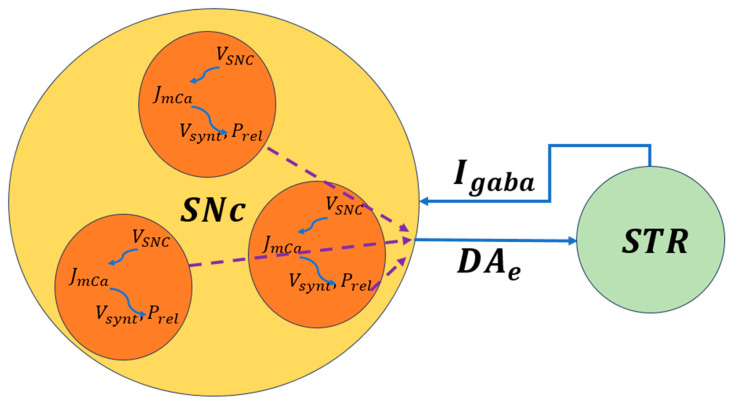
Schematic representation of bidirectional signaling between the SNc and striatum. Dopamine release, reuptake, and degradation regulate corticostriatal plasticity and BG output [50]. $V_{SNC}$, the voltage membrane of SNc neuron; $J_{mCa}$, the calcium flux of SNc neuron as a function of $V_{SNC}$; $V_{synt}$, the dopamine synthesis flux as a function of calcium; $P_{rel}$, the probability release of dopamine extracellularly as a function of calcium. The dotted lines represent the outputs of each cell combined to produce the extracellular dopamine.

The biophysical models of SNc further delve into molecular-level mechanisms including metabolic vulnerabilities, oxidative stress, calcium dysregulation, and alpha-synuclein aggregation that contribute to neuronal death. A simplified illustration of molecular-level interactions between the SNc and striatum is shown in Figure 4, where dopaminergic signaling both shapes and is shaped by BG activity. Striatal feedback influences SNc firing and dopamine release, creating a dynamic coupling between circuit activity and cellular stress. This bidirectional interaction provides a mechanistic basis for understanding how altered motor demands and network states may accelerate dopaminergic degeneration in Parkinson’s disease.

#### 2.3.4. Pharmacological Interventions and Levodopa (L-DOPA) Therapy

While previous subsections focused on cellular and biophysical modeling of SNc degeneration and its impact on dopamine signaling (Figure 3 and Figure 4), these frameworks can be extended to incorporate therapeutic interventions. Pharmacokinetic–pharmacodynamic models provide a quantitative tool to simulate L-DOPA therapy, the clinical gold standard for Parkinson’s disease. Pharmacokinetic models describe L-DOPA absorption, distribution, metabolism, and clearance, while pharmacodynamic models capture its effects on dopamine synthesis, vesicular storage, release, and receptor activation at synaptic terminals [55,61,62,63].

**Figure 5:**
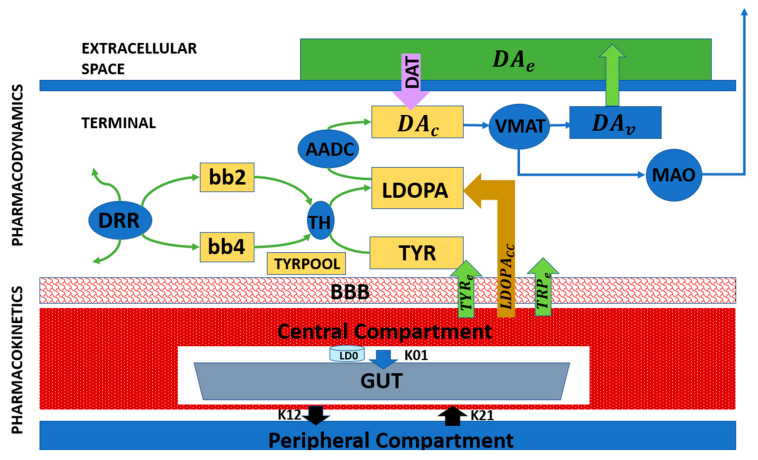
Block diagram representing the pharmacological model of L-DOPA administration [50].

Figure 5 presents a block diagram of a pharmacokinetic–pharmacodynamic model that can be integrated with cellular degeneration frameworks to capture therapeutic effects quantitatively. This integrated approach enables the simulation of how L-DOPA affects dopamine dynamics, network activity, and motor behavior over time. It also allows the investigation of motor fluctuations, long-term outcomes, and personalized treatment strategies, providing a mechanistic and predictive tool for clinical translation [55,63].

Extending cellular-level models in this way not only captures the mechanisms underlying progressive dopaminergic degeneration but also allows for the evaluation of how pharmacological interventions interact with residual neural circuits. When combined with behavioral and circuit-level models, pharmacokinetic–pharmacodynamic frameworks form a critical component of multiscale computational approaches to Parkinson’s disease, linking molecular pathology, neuronal vulnerability, network dysfunction, and therapeutic modulation. This integrated perspective naturally sets the stage for multiscale modeling, in which the cellular, circuit, and behavioral levels are unified to capture disease progression and response to treatment.

#### 2.3.5. Multiscale Approaches to PD Pathophysiology

By linking cellular degeneration mechanisms to larger circuit-level models of BG circuitry, cellular models of dopaminergic loss enhance our understanding of how dopamine depletion affects network dynamics at the level of individual neurons and populations. These models provide a mechanistic framework for studying molecular pathology, neuronal vulnerability, and system-level dysfunction, forming a critical component of multiscale computational approaches to PD. The combination of biophysically grounded cellular models with network-level simulations enables the personalized modeling of PD pathology and offers important insights into the mechanisms that drive disease progression and therapeutic responses.

Although cellular and molecular models of dopaminergic degeneration have primarily been evaluated in the context of limb motor behaviors, their implications naturally extend to oculomotor control through shared dopaminergic and basal ganglia gating mechanisms. Progressive calcium dysregulation, mitochondrial stress, and impaired dopamine synthesis in SNc neurons are expected to reduce striatal dopamine availability, thereby altering cortico–basal ganglia decision thresholds and inhibitory control. At the behavioral level, such changes would be expected to manifest as increased saccade latency, elevated antisaccade error rates, and reduced smooth pursuit gain—phenotypes commonly observed in Parkinson’s disease.

Importantly, direct empirical and computational mappings between molecular pathology and eye movement deficits remain limited, and this represents a critical direction for future work. Establishing predictive links across scales will require longitudinal validation and well-constrained priors, particularly as disease progression and therapeutic interventions unfold over time. In this context, eye movement measures provide highly quantifiable, low-dimensional behavioral outputs that are well suited to anchoring multiscale models and testing how molecular and biophysical processes of neurodegeneration translate into circuit dysfunction and observable behavior. Linking these measures with cellular and therapeutic models offers a promising pathway toward the development of more predictive and translational computational frameworks.

## 3. Translational and Clinical Applications

Beyond mechanistic understanding, computational and mathematical models are increasingly shaping therapeutic development and clinical decision-making in PD. By integrating biological data across scales, these models support rational drug design, individualized treatment strategies, and the optimization of neuromodulatory interventions. Importantly, modeling enables in silico experimentation that complements and, in some cases, precedes costly and time-consuming clinical trials.

### 3.1. Drug Discovery and Personalized Medicine

Mathematical modeling has become an integral component of modern drug discovery pipelines. Systems biology approaches integrate genomic, transcriptomic, proteomic, and metabolic data to identify dysregulated pathways and prioritize therapeutic targets. In PD, such models have been used to investigate pathways related to mitochondrial dysfunction, oxidative stress, α-synuclein aggregation, and calcium-dependent neurotoxicity, offering insights into potential disease-modifying strategies.

Pharmacokinetic and pharmacodynamic modeling is particularly impactful in PD, where L-DOPA therapy must balance symptomatic relief against motor complications such as dyskinesia and wearing-off effects. Pharmacokinetic and pharmacodynamic frameworks quantitatively link dosing regimens to synaptic dopamine levels and motor outcomes, enabling the systematic exploration of therapeutic windows. Individualized parameter estimation using patient-specific data represents a practical step toward personalized medicine, allowing dose schedules to be optimized based on disease stage, metabolic differences, and treatment history.

More broadly, model-based approaches offer a means to predict treatment failure, identify responder subgroups, and optimize combination therapies. While most progress has been made in PD, similar strategies are increasingly being explored in other neurodegenerative disorders, highlighting the generalizability of computational medicine.

### 3.2. DBS and Neuromodulation

DBS is one of the most successful surgical therapies for PD and represents a paradigmatic example of computationally informed intervention. Circuit-level models of the BG–thalamocortical network have demonstrated that high-frequency stimulation alleviates motor symptoms by disrupting pathological synchrony and beta-band oscillations, rather than simply suppressing neural activity [45,46].

Patient-specific DBS models now enable simulation of electrode placement, stimulation amplitude, pulse width, and frequency prior to surgery. By modeling current spread and network-level effects, these tools provide a mechanistic insight into how stimulation parameters influence both therapeutic efficacy and side-effect profiles. Such approaches are increasingly used to guide surgical planning and postoperative parameter tuning.

Increasing evidence further indicates that the clinical effects of DBS depend on the specific axonal pathways engaged by stimulation rather than on local neuronal inhibition alone. For example, recent work has shown that vestibular motion perception in PD patients correlates with the white matter tracts activated by STN DBS, highlighting the importance of pathway-specific stimulation effects and individual anatomical variability [64,65,66,67]. These findings reinforce the need for connectivity-aware, patient-specific computational models to predict stimulation spread and network-level outcomes.

In addition to motor outcomes, eye movement behavior has emerged as a sensitive functional readout of the effects of DBS on BG and brainstem circuitry. Because eye movements rely on well-defined cortico–BG–brainstem circuits and exhibit rapid, quantifiable dynamics, they provide an ideal behavioral domain for testing RL and decision-making models in Parkinson’s disease. STN DBS has been shown to modulate saccade latency, velocity, and inhibitory control in antisaccade tasks, reflecting alterations in oculomotor decision thresholds and action suppression mechanisms. These effects highlight the involvement of BG outputs to the superior colliculus (SC) and frontal eye field (FEF) circuits, which overlap with pathways implicated in motor and cognitive control. Computational modeling of DBS-induced changes in oculomotor behavior therefore provides a tractable framework for linking stimulation parameters, pathway engagement, and behavioral outcomes, and suggests that eye movement metrics may serve as valuable biomarkers for optimizing stimulation settings [29,30,64,65,66,67].

A major frontier in DBS research is the development of adaptive or closed-loop stimulation systems. In these paradigms, stimulation parameters are dynamically adjusted based on real-time neural biomarkers, such as beta-band power or phase-locked activity. Designing such feedback-driven neuromodulation strategies would be nearly impossible without computational frameworks that link neural signals to behavioral outcomes and control policies. Modeling thus plays a central role in advancing DBS from an open-loop intervention to a precise, responsive therapy.

### 3.3. Biomarkers and Early Diagnosis

One of the most promising applications of computational modeling lies in biomarker discovery and early diagnosis. Model-based analyses of motor behavior have demonstrated that kinematic features such as gait variability, turning dynamics, and obstacle negotiation can reveal disease-related impairments not captured by traditional clinical scales. Computational studies of gait in constrained environments, such as navigating narrow doorways, illustrate how task-specific motor deficits emerge from altered BG dynamics [68]. Similarly, experimental modeling approaches have been proposed to improve the assessment of Parkinsonian motor symptoms by linking observed behavior to latent computational variables governing action selection and vigor.

Machine learning and hybrid mechanistic–statistical models integrate multimodal data—including neuroimaging, electrophysiology, kinematics, speech, handwriting, and gait—to identify subtle disease signatures that precede overt motor symptoms [69,70]. Among these modalities, eye movement behavior offers a uniquely sensitive and mechanistically interpretable window into BG dysfunction in Parkinson’s disease.

Eye movements are rapid, highly stereotyped, and supported by well-characterized cortico–BG–brainstem circuits, making them particularly amenable to quantitative analysis and computational modeling [4,29]. Patients with PD exhibit robust and reproducible oculomotor abnormalities, including increased saccade latency, reduced saccade velocity, hypometric saccades, impaired smooth pursuit gain, and deficits in antisaccade performance [30,71]. These features reflect impairments in action initiation, inhibitory control, and reward-dependent decision thresholds—core computational processes directly modulated by dopamine [12,72]. Importantly, several oculomotor measures show sensitivity to early or prodromal disease stages and demonstrate responsiveness to dopaminergic medication and DBS, highlighting their potential utility as both diagnostic and treatment-monitoring biomarkers [73,74].

Although eye movement abnormalities provide a mechanistically grounded source of quantitative biomarkers, most oculomotor measures should currently be regarded as research-stage candidate markers rather than clinically validated diagnostic tools. Their primary value lies in their sensitivity to BG dysfunction and their interpretability within computational frameworks, where they can serve as quantitative phenotypes for model validation and hypothesis testing. Their translation to routine clinical use will require large-scale longitudinal validation, assessment of disease-stage sensitivity, and demonstration of added value beyond existing clinical scales.

Computational models provide a principled framework for linking these observable eye movement features to latent neural and algorithmic variables, such as RPE/dopamine equivalent clamp/limit values, decision thresholds, exploration parameter, and BG pathway balance [75,76]. For example, increased saccade latency and antisaccade error rates can be interpreted as shifts in action selection thresholds under dopaminergic depletion, while abnormalities in smooth pursuit dynamics may reflect altered sensory prediction and feedback integration [31,32]. By grounding eye movement biomarkers in mechanistic models, it becomes possible to distinguish disease-related changes from compensatory strategies and inter-individual variability, thereby strengthening their interpretability and translational relevance. Table 2 summarizes commonly studied eye movement biomarkers in Parkinson’s disease, their underlying neural and computational interpretations, and their relevance for early diagnosis, disease progression, and therapeutic monitoring.

Longitudinal modeling further enables the tracking of disease progression and differentiation between phenotypic subtypes. The long-term vision is a unified digital biomarker ecosystem in which disease risk, progression, and treatment response can be monitored continuously and noninvasively, transforming both clinical practice and clinical trials.

## 4. Challenges and Future Directions

Despite substantial progress, important limitations continue to constrain the predictive power and clinical translation of computational models of PD. Rigorous validation against longitudinal, multimodal datasets and evaluation across independent cohorts remain essential for assessing the generalizability and reproducibility of model-derived predictions. While many models provide compelling mechanistic explanations, their quantitative validation and generalizability remain uneven across domains. Parameter uncertainty and inter-individual variability constrain predictive accuracy, particularly when models are applied beyond the conditions under which they were developed.

At the circuit level, STN–GPe models successfully reproduce qualitative features of pathological beta-band activity, including the relevant frequency range, burst-like dynamics, and sensitivity to dopaminergic state or stimulation. However, quantitative agreement with patient local field potential recordings—such as beta power distributions, burst durations, and inter-individual variability—varies across cohorts and experimental conditions. While a small number of studies explicitly distinguish lower and higher beta sub-bands, as well as interactions with theta, alpha, or gamma activity, systematic quantitative validation against large patient datasets remains limited. As a result, current validation is primarily qualitative rather than fully quantitative, limiting the ability of these models to generate precise patient-specific predictions.

At the behavioral level, reinforcement learning-based models capture group-level effects of dopaminergic medication on learning asymmetries, action selection, and movement vigor. Nonetheless, predictive accuracy at the level of individual patients remains limited due to parameter uncertainty, task dependence, and heterogeneity in disease phenotype. Although medication effects and response curves can be qualitatively reproduced by tuning model parameters, current studies do not establish longitudinal, patient-specific mappings between empirical response trajectories and inferred model parameters. Consequently, RL models currently serve best as explanatory and hypothesis-generating frameworks rather than individualized predictors of treatment response.

Pharmacokinetic and pharmacodynamic models of levodopa therapy provide valuable quantitative descriptions of dopamine synthesis, release, and receptor activation, but rely on simplifying assumptions that limit their clinical applicability. Many models assume time-invariant pharmacokinetic parameters, homogeneous residual dopaminergic terminals, and a single smooth effect-site dopamine signal driving motor output. These assumptions neglect clinically important sources of variability, including disease progression, repeated dosing effects, non-dopaminergic deficits, task-dependent motor demands, and real-world pharmacokinetic fluctuations arising from gastric emptying, dietary amino acid competition, and gastrointestinal dysfunction. Addressing these limitations will be essential for improving the translational utility of pharmacokinetic and pharmacodynamic simulations.

In this context, eye movement measurements offer a promising complementary data source for model validation and parameter constraint. Oculomotor behaviors such as saccade latency, antisaccade error rates, and smooth pursuit gain can be measured with high temporal precision, minimal task demands, and strong cross-site reproducibility. These properties make eye movements particularly well suited to the longitudinal validation of computational models and to constraining individual-level parameters across disease stages and treatment conditions.

More broadly, a key challenge lies in bridging mechanistic and data-driven approaches. Many biologically grounded models and machine learning frameworks currently exist in isolation. Developing transparent hybrid models that combine interpretability with predictive performance will require standardized data formats, open benchmarking efforts, and rigorous validation against longitudinal multimodal clinical datasets.

Translation into clinical practice remains another major hurdle. Computational insights often fail to influence treatment decisions because models are not embedded within clinical workflows, lack regulatory alignment, or do not produce outputs that are actionable for clinicians. Addressing these barriers will require sustained collaboration between computational scientists, experimentalists, clinicians, and regulatory bodies.

Key future directions for computational modeling in PD include:

- Development of digital twins that integrate multimodal patient data, including eye-tracking metrics (saccade latency, antisaccade error rates, smooth pursuit gain), local field potentials, structural and diffusion MRI, postoperative CT imaging, and pharmacokinetic profiles, within Bayesian or hierarchical modeling frameworks for individualized parameter inference and disease tracking. Neuroimaging data are used to constrain model structure and prior distributions, including network topology, coupling strengths, dopaminergic integrity, and stimulation geometry, thereby enabling individualized parameter inference rather than serving as direct behavioral predictors. Within this framework, computational models comprise latent parameters such as learning rates, dopamine gain, exploration–exploitation balance, decision thresholds, coupling gains, time constants, and bias terms, which can be systematically tuned to derive behavioral metrics.
- Integration of eye-movement-based digital biomarkers into multiscale computational models to provide non-invasive, high-temporal-resolution readouts of BG function, decision-making dynamics, and disease progression, and to constrain latent model parameters at the individual level.

For example, diffusion MRI-derived connectivity constrains priors over BG–thalamocortical coupling parameters, postoperative CT imaging defines stimulation volumes used to parameterize electric-field models affecting STN neuronal populations, and local field potential beta-band features inform likelihood functions or optimization targets over synaptic gain and time-constant parameters.

In practice, parameter inference within such digital twin and multiscale modeling frameworks is achieved using a range of complementary strategies. Depending on data availability and model complexity, latent parameters may be estimated through optimization-based fitting, iterative trial-and-error tuning, simulation-based approaches, or data-driven methods such as neural networks and multilayer perceptron, in addition to Bayesian or likelihood-free inference. These approaches typically minimize discrepancies between simulated outputs and observed behavioral, electrophysiological, or clinical measurements, rather than computing closed-form posterior distributions.

Accordingly, future computational models of PD should be explicitly designed to support rigorous longitudinal validation. In this context, longitudinal validation refers to evaluating whether model parameters inferred at one time point reliably predict within-subject behavioral, electrophysiological, or clinical changes at subsequent measurements. This includes assessing parameter stability when physiological states are unchanged, systematic parameter drift with disease progression, and sensitivity to therapeutic interventions such as dopaminergic medication or DBS. Crucially, longitudinal validation emphasizes temporal prediction rather than repeated cross-sectional fitting, thereby distinguishing true disease-related dynamics from noise or overfitting. This concept is distinct from generalizability across cohorts and reproducibility of inference pipelines, which address population-level transfer and methodological robustness, respectively.

- Advancement of closed-loop DBS systems guided by biomarker signals beyond mean beta power, including beta burst duration, phase–amplitude coupling, gamma-band activity, oculomotor reaction times, antisaccade performance, and fixation stability. Recent adaptive DBS studies employing multi-site BG recordings and real-time control strategies demonstrate the feasibility and therapeutic promise of such approaches [84,85].
- Additional spectral features, such as relative band power and cross-frequency interactions, may further enrich control signals, although their clinical relevance remains to be established through systematic validation.
- The development of hybrid mechanistic–data-driven models that combine biologically grounded BG circuit dynamics with machine learning components to improve the prediction of individual treatment response while retaining interpretability.
- The generation of large-scale, interoperable computational platforms enabling longitudinal validation, cross-cohort reproducibility, and clinical translation of model-derived biomarkers.

## 5. Conclusions

Neurodegenerative diseases such as PD represent one of the most enduring challenges in modern medicine, arising not from isolated failures but from progressive dysfunction across interacting molecular, cellular, circuit, and behavioral systems. Mathematical and computational modeling is uniquely positioned to confront this complexity by integrating diverse biological processes into coherent, quantitative frameworks capable of linking mechanism to behavior.

This review has synthesized computational models of PD spanning behavioral, circuit, and cellular levels, highlighting how mechanistic approaches can bridge dopaminergic pathology, BG dynamics, and observable motor and cognitive phenotypes. Rather than advocating for a single unifying model, we emphasize how complementary modeling frameworks capture distinct facets of disease pathophysiology and together advance mechanistic understanding.

As discussed in the challenges and future directions, a central frontier for the field lies in enhancing the predictive power of computational models through rigorous longitudinal validation. Models must not only reproduce cross-sectional effects but also demonstrate stability, sensitivity, and temporal predictive accuracy within individuals across disease progression and therapeutic interventions. Achieving this goal requires well-constrained priors informed by biologically grounded and quantitatively reliable data sources.

Within this multiscale framework, eye movement behavior exemplifies how tightly controlled, high-temporal-resolution behavioral assays can serve as scalable, non-invasive readouts of BG function and decision-making dynamics. When integrated into computational models, such measures offer a powerful means to constrain individual-level parameters and to link neural dynamics with clinically relevant outcomes over time.

Ultimately, the goal of computational modeling in PD is translational: to support biomarker discovery, therapy optimization, and personalized clinical decision-making. What began as abstract theoretical neuroscience has matured into a discipline with growing clinical relevance. As data availability, computational methods, and interdisciplinary collaboration continue to expand, computational models are poised to function not merely as explanatory tools but as predictive engines guiding precision interventions. In this vision, modeling is no longer ancillary to experimentation—it becomes an equal partner in the effort to understand, monitor, and treat Parkinson’s disease.

## Figures and Tables

**Table 1:** Illustrative mapping of RL elements, corresponding neural substrates, and their functions.

RL Element	Neural Substrate	Function
State	Sensorimotor cortex	Represents task state
Action	Motor cortex + BG-thalamus loop	Generates movement
Policy	D1/D2 pathways, corticostriatal synapses	Action selection strategy
Reward & TD Error	Dopamine system (SNc/VTA)	Feedback and learning signal
Exploration	STN–GPe loop + cortical noise	Motor variability for exploration

**Table 2:** Eye movement-based biomarkers in PD [4,12,29,30,71,72,77,78,79,80,81,82,83].

Eye Movement Measure	PD-Related Alteration	Computational Interpretation	Neural Substrate	Clinical Relevance	Sensitivity to Disease Stage/Treatment
Saccade latency	Increased (mild for visually guided and strong for voluntary saccades)	Elevated decision threshold/stronger tonic inhibition, weaker cortical drive	BG to SC disinhibition (tonic SNr/GPi), cortical drive (FEF/LIP)	Candidate marker of processing speed/executive load	Increases with disease progression; reduced by dopaminergic medication and STN DBS
Antisaccade performance (error rate and latency)	Increased direction errors and latency	Weaker inhibitory control (NoGo/indirect efficacy), weaker hyper direct	Top-down control (DLPFC/FEF) + BG gating + SC suppression	Candidate Marker for inhibitory control and executive dysfunction	Sensitive to early cognitive involvement. Modulated by dopaminergic medication and DBS
Saccade gain (hypometria)	Reduced. Hypometria/multi-step corrections	Deficient gain control. (SC to displacement mapping)	BG-SC selection + brainstem burst generator; cerebellar calibration contributes to accuracy	Candidate marker for oculomotor bradykinesia	Present early; partially responsive to medication; variably affected by DBS
Peak velocity/main sequence	Often near-normal in idiopathic PD; some cohorts show reductions	Reduced burst drive or delayed/partial disinhibition	Brainstem burst generator + Omni pause; SC drive; BG influences via SC release	Best used for differential diagnosis and mechanistic phenotyping	Often preserved in idiopathic PD; reduced in atypical Parkinsonism
Smooth pursuit gain	Reduced gain, more catch-up saccades	Altered sensory prediction & feedback integration	Cortico–pontine–cerebellar circuit	Promising research-stage marker for sensorimotor integration	Impaired early; improves with medication; sensitive to disease severity
Fixation stability/microsaccades	Altered/Increased fixation instability	Higher oculomotor noise/weaker fixation attractor stability	SC + cortical attention networks	Emerging research stage biomarkers, not yet established as clinical standard	Sensitivity under investigation; altered by DBS and attentional state

## Data Availability

No new data were created or analyzed in this study.

## Abbreviations

The following abbreviations are used in this manuscript:

PD	Parkinson’s Disease
SNc	Substantia Nigra pars Compacta
BG	Basal Ganglia
RL	Reinforcement Learning
STN	Subthalamic Nucleus
GPe	Globus pallidus externa
DBS	Deep Brain Stimulation
RPE	Reward Prediction Error
GEN	Go-Explore-NoGo
VTA	Ventral Tegmental Area
ROS	Reactive Oxygen Species
L-DOPA	Levodopa
FEF	Frontal Eye Field
LIP	Lateral Intraparietal Area
DLPFC	Dorsolateral prefrontal cortex
SC	Superior Colliculus
